# Circular PVT1 regulates cell proliferation and invasion via miR-149-5p/FOXM1 axis in ovarian cancer

**DOI:** 10.7150/jca.52234

**Published:** 2021-01-01

**Authors:** Min Li, Chi Chi, Liqin Zhou, Youguo Chen, Xiuwu Tang

**Affiliations:** 1 Department of Gynecology & Obstetrics, the First Affiliated Hospital of Soochow University, Suzhou 215006, Jiangsu Province, China.; 2 Department of Gynecology & Obstetrics, Suzhou Xiangcheng People's Hospital, Suzhou 215006, Jiangsu Province, China.

**Keywords:** PVT1, ovarian cancer, bioinformatics, circRNA, FOXM1

## Abstract

Long non-coding RNA plasmacytoma variant translocation 1 (PVT1) is a dysregulated gene in malignancy and is associated with oncogenesis. In this study, we found PVT1 RNA was an ovarian specific expressing gene, and overexpressed in multiple cancer types, including ovarian cancer (OV). Higher expression levels of PVT1 are related to shorter survival time in OV patients, especially in patients with advanced stage and grade. Recent studies indicated circular PVT1 also had an important role in cancer progression, whose roles in OV remain unclear. Knockdown of circular PVT1 significantly suppressed OV cell proliferation, migration and invasion. Bioinformatics analysis demonstrated that circular PVT1 was involved in regulating angiogenesis, osteoblast differentiation, regulation of cell growth, type B pancreatic cell proliferation, negative regulation of apoptotic process, phospholipid homeostasis, regulation of neurogenesis, definitive hemopoiesis, cell migration, regulation of glucose metabolic process, central nervous system development and type 2 immune response. Our data showed miR-149-5p targeted FOXM1, which was regulated by circular PVT1. Forkhead Box M1 (FOXM1) expression in ovarian cancer exhibited high level when compared with normal tissues, and had relation with relatively poor survival. FOXM1 promoted cell viability and reduced FOXM1 could rescue circular influence of circular PVT1-caused carcinoma induction. In conclusion, circular PVT1 increased FOXM1 level via binding to miR-149-5p and thus affected ovarian cancer cell viability and migration.

## Introduction

In 2018, the incidence rate of ovarian cancer was 6.6/100000 all over the world 1. Ovarian cancer-induced morbidity and related fatality ratio in women ranked the top 2. The occurrence proportion of ovarian cancer varies largely from one geography area and ethnic population to another, especially was higher in Northern Europe and United States but lower in Japan 2. The morbidity of ovarian cancer is the third highest, while the mortality rate is the first in overall gynecological malignancies 2. The global occurrence ratio of ovarian cancer is 3.6%, and death ratio is as high as 4.3% 3. The pathogeny involved in ovarian cancer, particularly epithelial ovarian cancer (EOC), stayed elusive. Snail 4 and Twist family 5, belonging to transcription factors, were reported to modulate the expression of E-cadherin and was shown to have association with progress of ovarian cancer. Non-coding RNAs comprising microRNAs (miRNAs) and lncRNAs probably displayed important roles in ovarian cancer 6, 7. Let-7 is considered to be a prognosticator of ovarian cancer 8, 9. Overexpression of miRNA-30a-5p induced ovarian cancer cell viability, migration, invasion and formation of colony 10. MiR-133a is abnormally expressed in various tumors, such as osteosarcoma, ovarian cancer, lung cancer, and esophageal cancer, gastric cancer, and so on 11. LncRNA-SNHG15 is an oncogene of ovarian cancer, which can induce EOC cell viability, migration and invasion 12. LncRNA plasmacytoma variant translocation 1 (PVT1) motivated progress of ovarian cancer via down-regulating miR-214 13, 14.

CircRNA occurs in splicing procedure of transcription, and single-stranded RNA molecules shape a circle through covalent bonds 15. Despite circRNA was found decades ago, it was originally thought to be from errors of RNA splicing 15. CircRNA had been demonstrated to have an important role in cancer cells. For example, circRNA-ITCH inhibits the expressions of miRNA-7, miRNA-17 and miRNA-214 in esophageal squamous cell carcinoma by sponge adsorption 16. CircRNA hsa_CircRNA_101996 induced cervical cancer cell viability and invasion by motivation of TPX2 which functioned as repressor of miR-8075^17^. Bioinformatics analysis indicated that circRNAs participated in metabolism of glucose, cell cycle of mitosis and production of ovarian steroid 18, 19. Circular RNA-HIPK3 increased the occurrence of EMT in ovarian cancer by activating miR-338-3p, followed by up-regulating HIF-1α 20. However, the roles of circRNAs in ovarian cancers remained unclear.

Our study revealed that circular PVT1 promoted the tumor progression in ovarian cancer. Our data have shown a new pathway circular PVT1 affected ovarian cancer cell viability and metastasis via miR-134-5p/FOXM1, thus providing a hint of uncovering novel countermeasure for ovarian cancer.

## Materials and methods

### Cell culture and transfection

SKOV3 and A2780 were derived from American Type Culture Collection (ATCC) and kept in RPMI-1640 medium supplied by 10% FBS (Thermo Fisher, USA) with penicillin (50 U/mL)/streptomycin (50 μg/mL) (Thermo Fisher, USA). All indicated siRNA and respective negative control (NC) were constructed by Shanghai Genepharma Company (China). CircPVT1-specific siRNAs and si-NC, as well as miR-149-5p mimics/inhibitor, were all designed by and obtained from Shanghai Sangon Biotech Corp. (Shanghai, China). 100 nM RNA oligonucleotides were transfected into indicated cells by LipofectamineTM 2000 (Invitrogen, Waltham, USA). All the transfected cells were collected at 48 h post-transfection for the following use.

### qPCR

The method of qPCR was described in previous studies. Internal controls for miR-149-5p and lncRNA/mRNA were U6 and GAPDH, respectively. The primers include: GAPDH: 5ʹ-CCAGGTGGTCTCCTCTGA-3' and 5ʹGCTGTAGCCAAATCGTTGT-3'; U6: 5ʹ-CTCGCTTCGGCAGCACA-3' and 5ʹAACGCTTCACGAATTTGCGT-3'; circPVT1: 5ʹ-GGTTCCACCAGCGTTATTC-3' and 5ʹ-CAACTTCCTTTGGGTCTCC-3'; miR-205-5p 5ʹ-ATTCTCTCATCTGGCTCCGTGT-3' and 5ʹ-TATGGTTGTTCTGCTCTCTGTGTC-3'.

### CCK-8 assay

2×10^5^ cells in each well were inoculated in 96-well plates. Medium was used as blank control. At post-hypoxiareoxygenation, indicated cells in per well were added 10 μL of CCK-8 (Beyotime Institute of Biotechnology, Beijing, China) solution, and kept at 37°C for 2 h. The OD value of 450 nm was detected. The final data was represented as cell viability, as previous study described 21.

### Luciferase assay

The sequences of wild-type (WT) or mutated (Mut) FOXM1 were purchased from Sangon (Shanghai, China) and then cloned into pGL3 construct (Promega Corporation, Madison, USA). MiR-149-5p mimics and corresponding reporter expression cassette were co-transfected into indicated cells by Lipofectamine 2000 as manual described. At 24 h post-transfection, relative firefly luciferase activity after normalized to renilla was detected by dual-luciferase reporter assay kit (Promega Corp.).

### Public datasets analysis

In this study, candidate miRNAs related to circPVT1 were forecasted by virtue of public bioinformatics tool Starbase (http://starbase.sysu.edu.cn/panCancer.php) and CircInteractome (https://circinteractome.nia.nih.gov/). A total of 9865 potential targets of circPVT1 were obtained. Then, we extracted different expressed genes in OV using GEPIA datasets. Finally, we constructed a circPVT1 associated ceRNA network using cystoscope software 22.

### Statistical analysis

Prism GraphPad 7.0 software was applied to analyze data in this literature. The presented data was shown as mean ± standard deviation (SD). Difference existing in two or multiple groups was assessed by one-way ANOVA. Significant difference means P value is not more than 0.05.

## Results

### PVT1 is overexpressed in ovarian tissues

**Figure 1:**
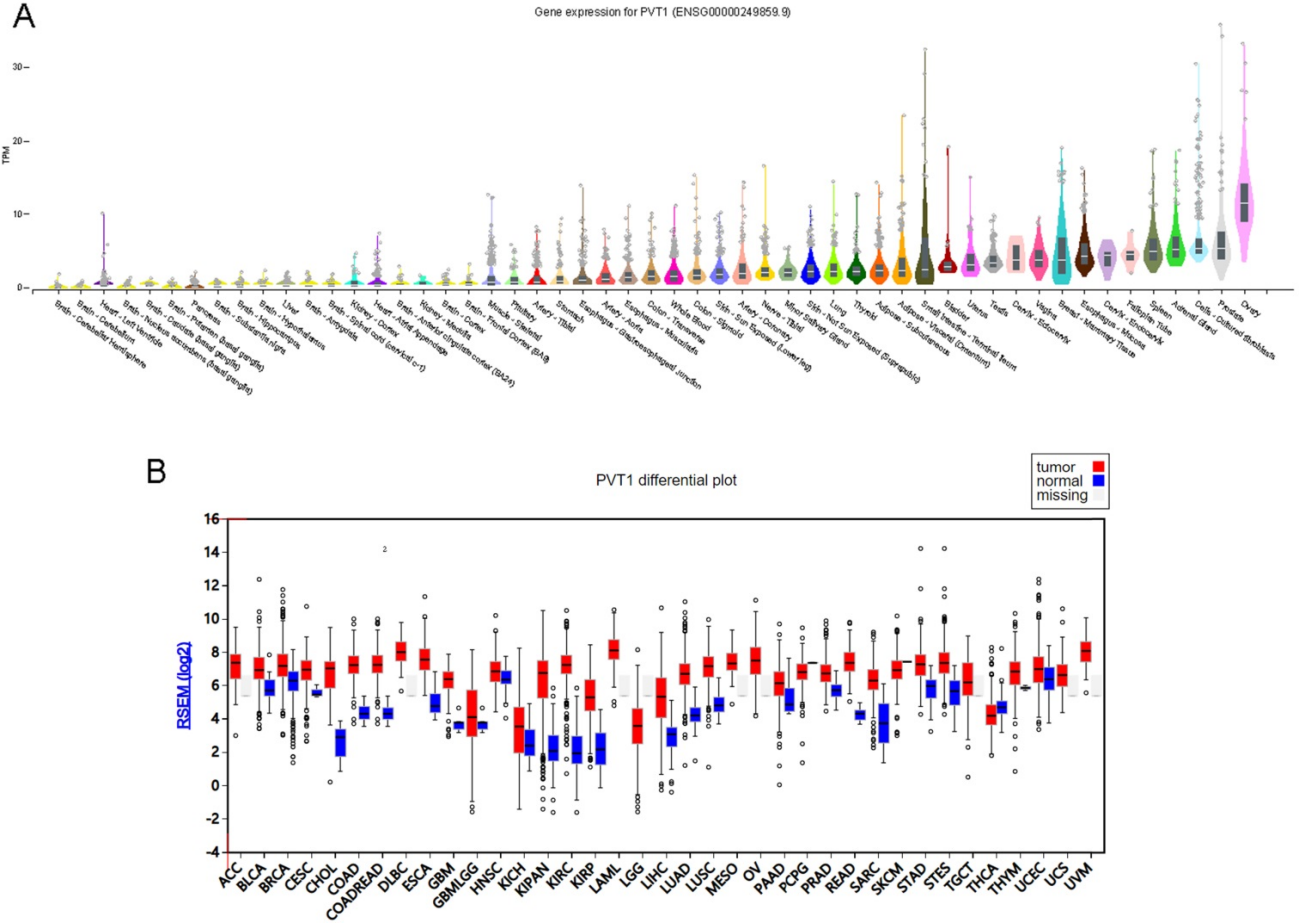
** PVT1 is an ovarian specific expressing gene.** (A) PVT1 expression patterns across human tissues using GTEx database. (B) PVT1 was differently expressed in human cancers.

We first screening PVT1 expression pattern across human tissues using GTEx database 23, 24. As shown in Figure 1, PVT1 was overexpressed in ovary tissues compared to other tissues, including prostate, adrenal gland, spleen, fallopian tube, cervix and brain tissues (Figure 1A).

Then, we detected whether PVT1 was differently expressed in human cancer. As present in Figure 1, the results showed that PVT1 was up-regulated in BLCA, BRCA, CESC, COAD, ESCA, GBM, KICH, KIPAN, KIRC, KIRP, LIHC, LUAD, LUSC, PRAD, STAD and STES. Very interestingly, we also observed PVT1 was overexpressed in OV compared to many types of normal tissues (Figure 1B).

### Higher expression level of PVT1 is correlated to poorer prognosis of ovarian cancer patients

**Figure 2:**
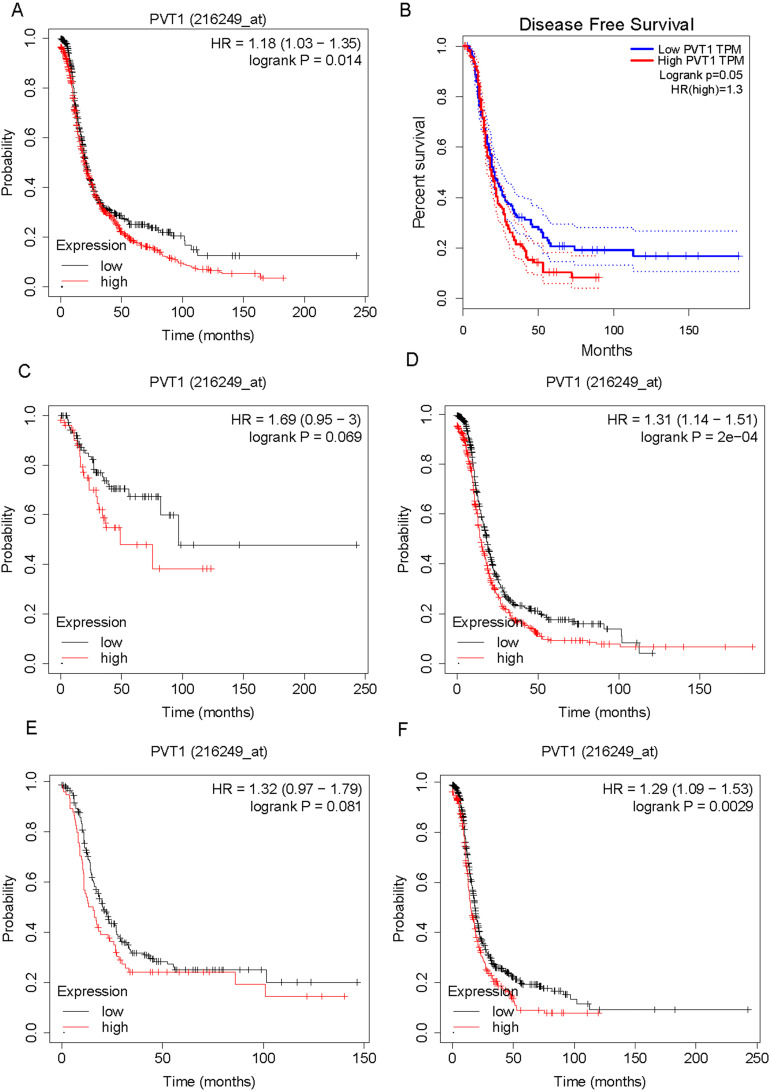
** Higher expression level of PVT1 mRNA is correlated to poorer prognosis of patients with ovarian cancer.** (A-B) higher expression of PVT1 mRNA level is correlated to shorter progression-free survival time in patients with OV by analyzing KM plotter database (A) and TCGA database (B). (C-D) The correlation between PVT1 mRNA levels and progression-free survival time in patients with high and low staged OV. (E-F) The correlation between PVT1 mRNA levels and progression-free survival time in patients with high and low graded OV.

We evaluated the correlation between PVT1 mRNA expression levels and survival time in OV patients. As shown in Figure 2, the results showed that higher expression of PVT1 mRNA correlates to shorter progression-free survival time in patients with OV by analyzing KM plotter database (Figure 2A) and TCGA database (Figure 2B). Then, we determined whether the correlation between PVT1 mRNA expression levels and survival time were affected by clinical parameters, such as grade and stage. Very interestingly, we found that higher expression of PVT1 mRNA correlates to shorter progression-free survival time in patients with stage 3/4 cancer (Figure 2C) and grade 3 OV (Figure 2E). However, we did not find a significant correlation between PVT1 mRNA expression levels and survival time in low staged (Figure 2D) and high-graded OV (Figure 2F).

### Circular PVT1 knockdown suppressed cell viability, migration and invasion

To investigate the biological roles of circPVT1 in OV, we performed loss-of-function experiments in OV cell lines. Compared to linear RNA, back-splicing site was the circRNA-specific sequence. SicircPVT1 was designed to target the back-splicing site (Figure (Figure 2A). Then, qPCR analysis revealed that circPVT1 level in si-circPVT1-1 treated groups was weaker than that in si-NC treated group, indicating successful transfection (Figure 5A and C). However, the expression level of lncRNA PVT1 was not affected after si-circPVT1-1 transfection in OV cells. In the following experiments, si-circPVT1-1 was chosen and named as si-circPVT1. Normalized cell viability in circPVT1-transfected SKOV3 and A2780 cells was obviously enhanced compared to that in NC-transfected group. In addition, normalized cell viability in si-circPVT1-transfected group was reduced than that in si-NC-transfected group (Figure 5B and D). Meanwhile, we found that knockdown of circular PVT1 significantly suppressed invasion (Figure 5E-G) and migration (Figure 5H-J) in SKOV3 and A2780.

### Bioinformatics analysis of circPVT1

**Figure 3:**
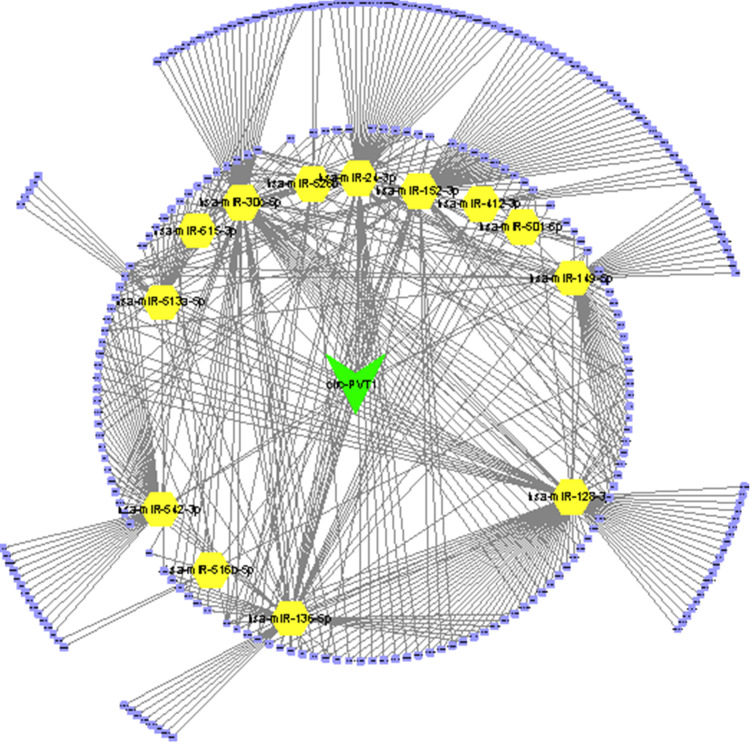
Construction of circPVT1 regulating ceRNA network.

Thus, we performed bioinformatics analysis to uncover the mechanisms of circPVT1 in OV. Previous studies revealed that circRNAs-mediated modulation of miRNA level via sponging miRNAs. To uncover whether circPVT1 sponged miRNAs regulate OV cell progress, candidate miRNAs related to circPVT1 were forecasted by virtue of public bioinformatics tool Starbase (http://starbase.sysu.edu.cn/panCancer.php) and CircInteractome (https://circinteractome.nia.nih.gov/). A total of 9865 potential targets of circPVT1 were obtained. Then, we extracted different expressed genes in OV using GEPIA datasets. Finally, we constructed a circPVT1 associated ceRNA network, which included 15 miRNAs (hsa-miR-30c-5p, hsa-miR-615-3p, hsa-miR-136-5p, hsa-miR-412-3p, hsa-miR-152-3p, hsa-miR-24-3p, hsa-miR-526b-5p, hsa-miR-516b-5p, hsa-miR-128-3p, hsa-miR-149-5p, hsa-miR-501-5p, hsa-miR-542-3p, hsa-miR-513a-5p) and 227 differently expressed mRNAs (Figure 3).

**Figure 4:**
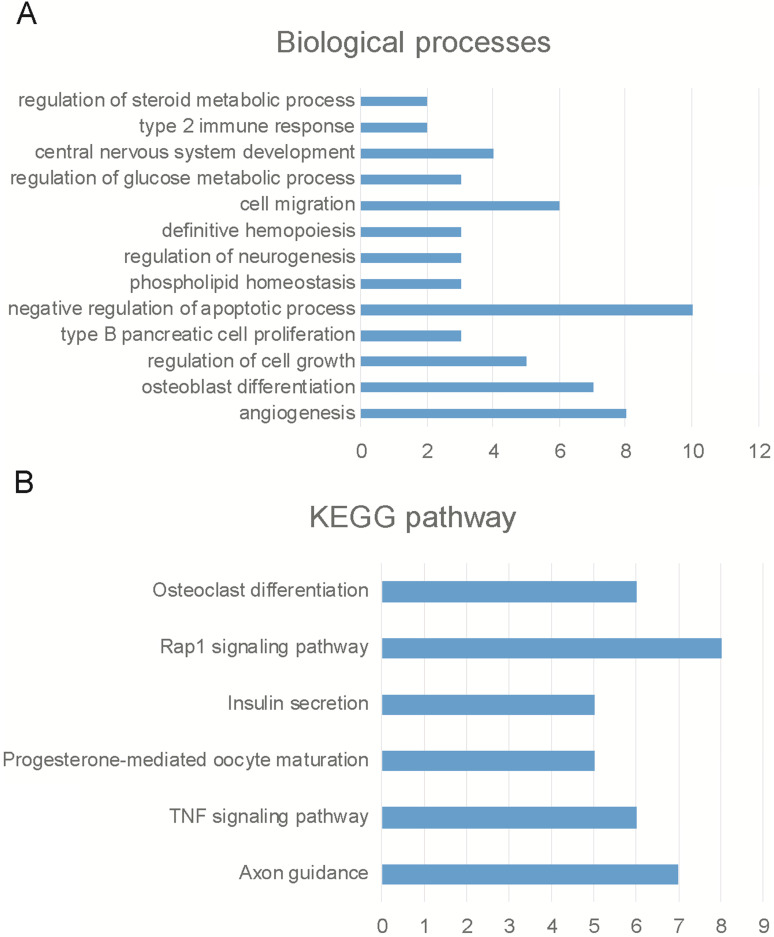
** Bioinformatics analysis of circPVT1.** (A) Bioinformatics analysis revealed circPVT1 regulating biological processes. (A) Bioinformatics analysis revealed circPVT1 regulating KEGG pathways.

**Figure 5:**
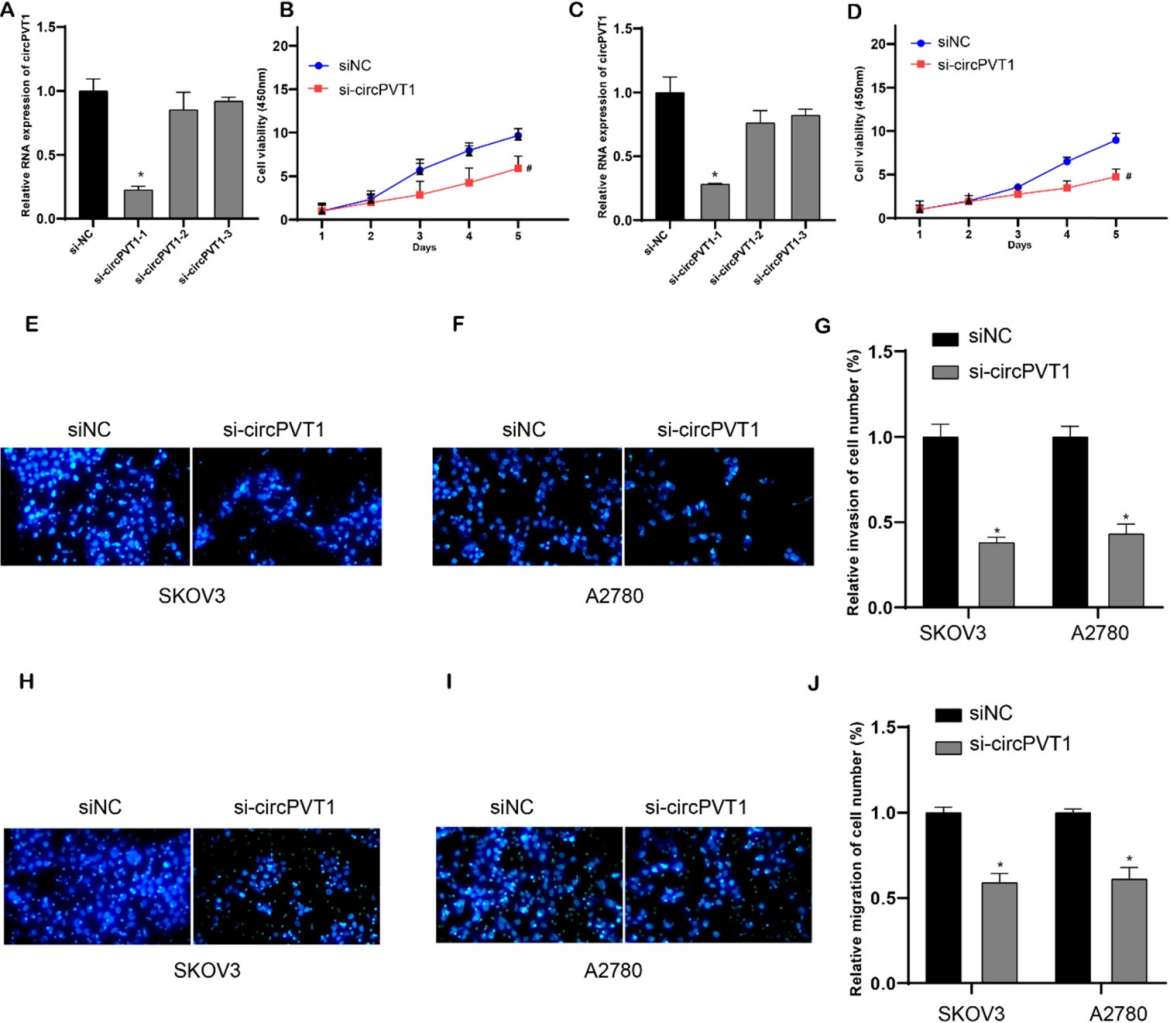
** Knockdown circular PVT1 suppressed cell viability, migration and invasion.** (A and C) qPCR analysis revealed that circPVT1 level in si-circPVT1-1 treated groups were weaker than that in si-NC treated groups in SKOV3 and A2780 cells. (B and D) CCK-8 analysis revealed that circular PVT1 knockdown suppressed SKOV3 and A2780 cell viability. (E-G) Transwell assay showed circular PVT1 knockdown suppressed SKOV3 and A2780 cell invasion. (H-J) Transwell assay showed circular PVT1 knockdown suppressed SKOV3 and A2780 cell migration.

Then, we employed bioinformatics analysis for this network. As presented in Figure 4, the results showed circPVT1 was involved in regulating angiogenesis, osteoblast differentiation, regulation of cell growth, type B pancreatic cell proliferation, negative regulation of apoptotic process, phosphorlipid homeostasis, regulation of neurogenesis, definitive hemopoiesis, cell migration, regulation of glucose metabolic process, central nervous system development and type 2 immune response (Figure 4A). KEGG pathway analysis showed circPVT1 was involved in regulating axon guidance, TNF signaling pathway, progesterone-mediated oocyte maturation, insulin secretion, rap1 signaling pathway, osteoclast differentiation, RAS signaling pathway and Jak-STAT signaling pathway (Figure 4B).

### Circular PVT1 targets miR-149-5p

**Figure 6:**
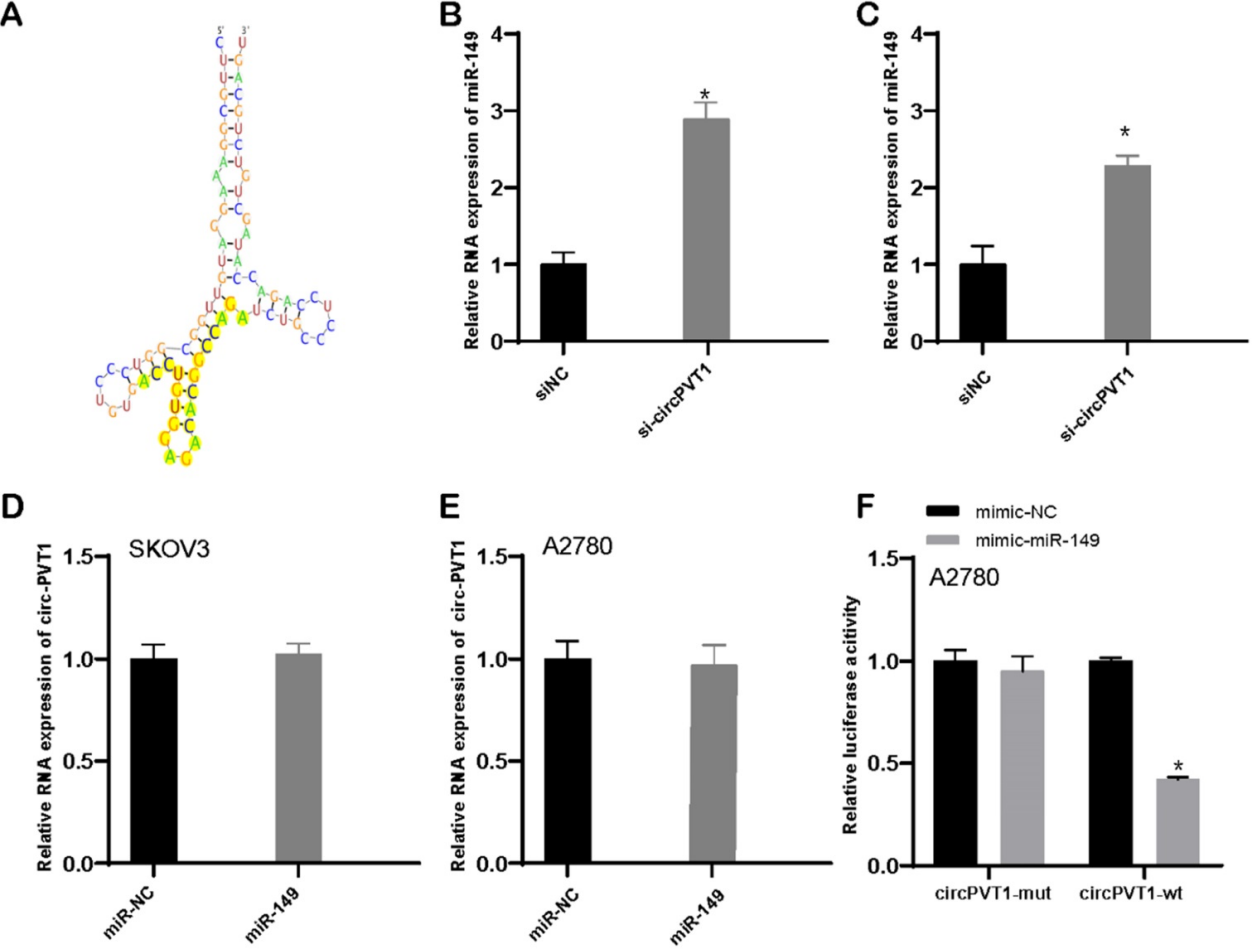
** Circular PVT1 targets miR-149-5p.** (A) Circular PVT1 targeted miR-149-5p. (B-C) miR-149-5p expression was detected after circular PVT1 knockdown. (D-E) Circular PVT1 expression was detected after overexpression of miR-149-5p. (F) The luciferase activity of luciferase reporter plasmid with circular PVT1 wild type fragment, not circular PVT1 mutant fragment was suppressed after overexpression of miR-149-5p.

Bioinformatics analysis revealed that circular PVT1 targeted miR-149-5p (Figure 6A). What is more, our data revealed that downregulated circular PVT1 enhanced miR-149-5p expression level (Figure 6B and C). Whereas overexpression of miR-149-5p exerted none influence on circular PVT1 (Figure 6D and E). Furthermore, we showed overexpression of miR-149 suppressed the luciferase activity of luciferase reporter plasmid with circular PVT1 wild type fragment, not circular PVT1 mutant fragment.

### MiR-149-5p targets FOXM1, a target of circular PVT1

**Figure 7:**
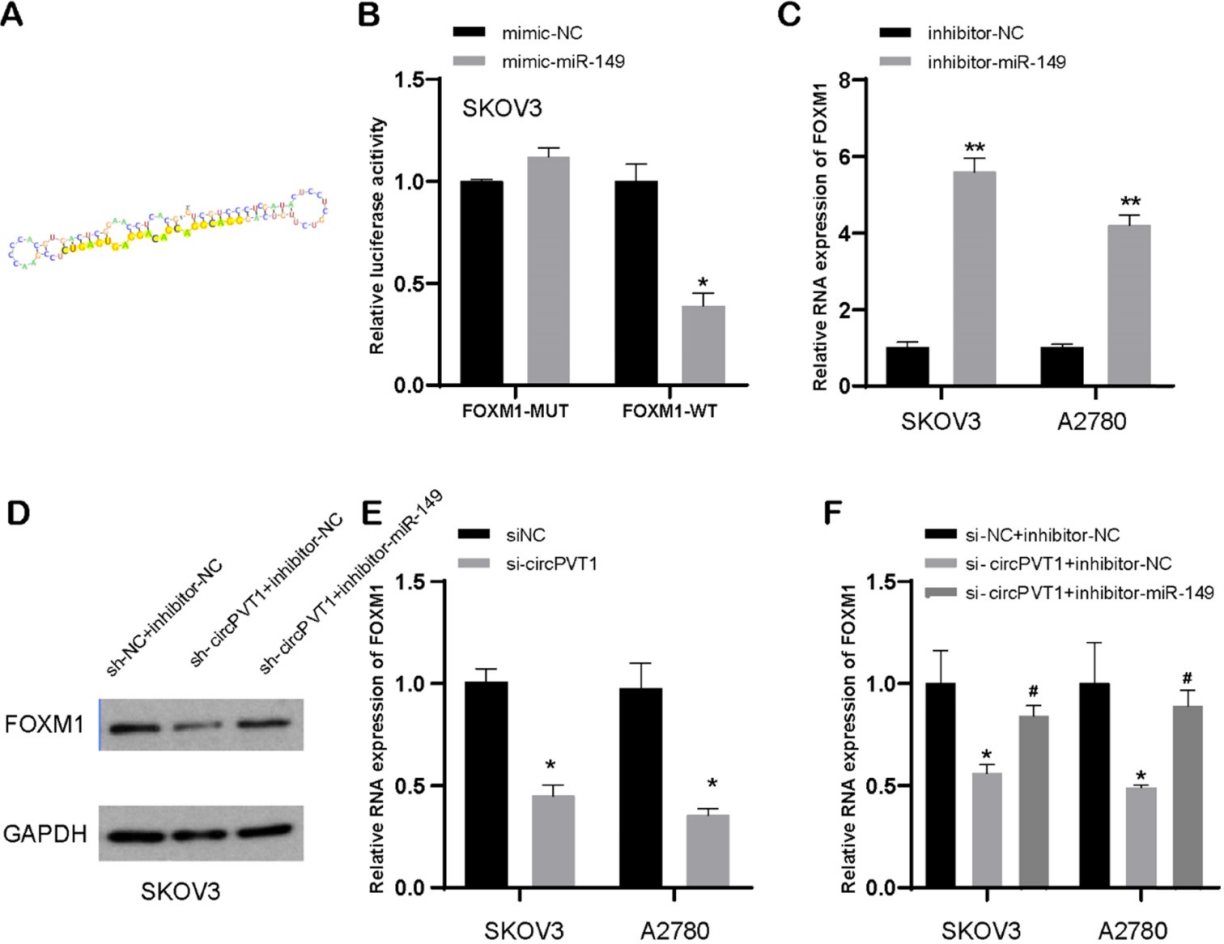
** MiR-149-5p targets FOXM1.** (A) MiR-149-5p targets FOXM1. (B) Luciferase experiment suggested that miR-149-5p bond to 3′-UTR region of FOXM1. (C) FOXM1 expression was detected after transfection with miR-149-5p inhibitors. (D) The protein level of FOXM1 was detected after transfection with NC, si-circPVT1 and miR-149-5p inhibitors. (E) FOXM1 expression was detected after transfection with si-circPVT1. (F) The mRNA level of FOXM1 was detected after transfection with NC, si-circPVT1 and miR-149-5p inhibitors.

TargetScan dataset was executed to search out targets of miR-149-5p (Figure 7A). Luciferase experiment suggested that miR-149-5p bond to 3′-UTR region of FOXM1 (Figure 7B). Subsequently, our data indicated that reduced miR-149-5p level resulted in upregulation of FOXM1 mRNA and protein level in SKOV3 and A2780 cells, compared to NC-transfected group (Figure 7C and D). Our data implied that FOXM1 was a direct target of miR-149-5p.

Additionally, our data showed that downregulated circular PVT1 could suppress FOXM1 expression in protein and mRNA levels (Figure 7D and E). Besides, our data also displayed that reduced circular PVT1 level could lead to ablated protein expression of FOXM1, while that effect could be reversed after knockdown of miR-149-5p to some extent (Figure 7D and F). This suggested that circular PVT1 modulated FOXM1 expression level after targeting miR-149-5p.

### FOXM1 expression had association with poor survival, and induced cell viability in ovarian cancer

**Figure 8:**
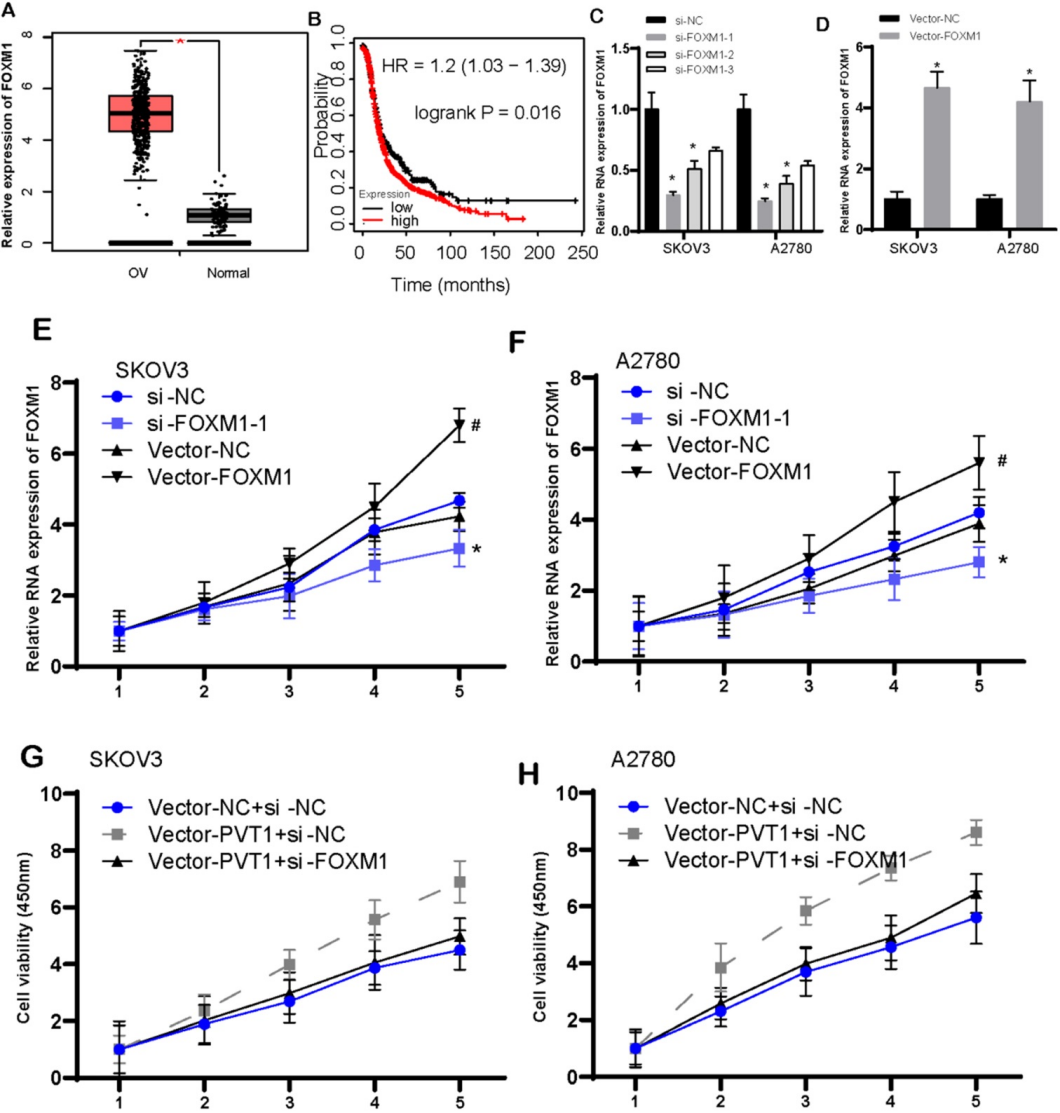
** FOXM1 expression had association with poorly prognostic status, and induced cell viability in ovarian cancer.** (A) FOXM1 was increased largely in OV tissues. (B) The correlation between FOXM1 levels and survival time in OV was analyzed. (C-D) The level of FOXM1 was detected after knockdown (C) and overexpressed (D) FOXM1 in the SKOV3 and A2780 cells. (E-F) Increased FOXM1 induced cell viability, however, reduced FOXM1 could cause inhibition of SKOV3 and A2780 cell viability. (G-H) Rescue assays revealed circular PVT1 modulates SKOV3 and A2780 proliferation via FOXM1.

Figure 8A illustrated FOXM1 was increased highly in OV tissues when compared with that in normal tissues. Ovarian cancer patients with high-expressed FOXM1 presented worse survival than those with low-expressed FOXM1 (Figure 8B). Then, we knockdown and overexpressed FOXM1 in the SKOV3 and A2780 cells (Figure 8C and D). Following data demonstrated that increased FOXM1 induced cell viability. However, reduced FOXM1 could cause inhibition of cell viability (Figure 8E and F). Our data implied that FOXM1 functioned importantly in promotion of tumor growth.

We also conducted rescue assays to validate circular PVT1 impeded tumor growth by targeting to FOXM1. Normalized cell viability in circPVT1+si-NC group was evidently stronger than that in circPVT1+NC group. However, the cell viability in circPVT1+sh-FOXM1 group was weaker than that in circPVT1+si-NC group (Figure 8G and H).

## Discussion

PVT1 is an extremely dysregulated gene in malignancy and is reported to have association with oncogenesis 25. PVT1 level is increased in multiple types of human cancer, comprising ovarian cancer 13, 14, breast cancer 26, 27 and non-small cell lung cancer 28, 29. PVT1 functioned importantly in promoting cancer viability, autophagy and metastasis 30. For example, C-Myc-promoted PVT1 induced cervical cancer cell growth by virtue of sponging miR-486-3p 31. LncRNA PVT1 mediated promotion of cervical cancer cell viability and invasion via increasing smad3 level, which was responsible for sponging miR-140-5p 32. In ovarian cancer, PVT1 was found to promote tumor progression by silencing miR-214 13. This study also showed PVT1 mRNA overexpression was correlated to shorter survival time in patients with OV. Here, our findings identified the function and mechanism of circPVT1 and FOXM1 involved in ovarian cancer, indicating circular PVT1-mediated regulation of cell apoptosis and metastasis by way of targeting miR-149-5p axis. We presumed our study would give a new hint of uncovering biomarker for ovarian cancer.

CircRNAs have been found to be a type of key regulators in cancers. It has been found that circular PVT1 is a carcinogenic non-coding RNA and has important clinical significance in numerous carcinoma including ovarian cancer, gastric cancer and thyroid cancer 33-42. For example, reduced circular RNA circPVT1 retarded gastric cancer growth via microRNA-3666-mediated reduced SIRT7 level 43. CircPVT1 functioned as a proliferation factor and therapeutic target in esophageal cancer 44. Enhanced circPVT1 accelerated oral squamous cell cancer growth via sponging miRNA 45 and circular RNA-PVT1-mediated metastasis of colon cancer via sponging miR-145 40. Circular RNA PVT1 as miR-497's competitive endogenous RNA boosted the progression of NSCLC 41. The carcinogenic effects of circPVT1 on head and neck squamous cell cancer were caused by mutated p53/YAP/TEAD transcriptional active complex 46. Multiple previous studies demonstrated that circPVT1 may act as miRNA sponge. For example, Circ-PVT1 regulates cell growth, metastasis and glycolytic metabolism of oral squamous cell carcinoma via miR-106a-5p/HK2 axis, promotes metastasis via regulating of miR-526b/FOXC2 signals in OS cells, enhances cell proliferation but inhibits apoptosis through sponging microRNA-149 in epithelial ovarian cancer, contributes to chemotherapy resistance of lung adenocarcinoma through miR-145-5p/ABCC1 axis. Nevertheless, there are few reports regarding circPVT1-induced effects on OV. Our data demonstrated circular PVT1 led to promotion of cell viability but inhibition of cell apoptosis. Reduced circular PVT1 lowered resistance to drug, revealing circular PVT1-mediated influence on ovarian cancer and the possibility of participating in drug resistance of ovarian cancer.

To deeply investigate the phenomenon of ovarian cancer cell viability and apoptosis induced by circular PVT1, we conducted bioinformatics analysis and found circular PVT1 regulated miR-149-5p in the form of ceRNA, and miR-149-5p directly bond to 3'UTR of FOXM1 mRNA to repress FOXM1 expression. MiR-149 is considered to be a versatile tumor suppressor, which is down-regulated in several cancer types 47-51. MiR-149-5p was identified to be a suppressor of ovarian cancer. For example, low-expressed miR-149 was related to poorly prognostic status, while high-expressed miR-149 heightened the sensitiveness of ovarian cancer cells when exposed to cisplatin. It is further proved that X-linked apoptosis inhibitory factor (XIAP) is a target of miR-149 and participates in miR-149-related role in ovarian cancer. LncRNAs could target MiR-149-5p and further regulate related genes' expression. In this study, miR-149 was reduced in tissues and cells of ovarian cancer. Our results implied that miR-149-5p was modulated by circular PVT1, thus inhibiting viability and apoptosis of ovarian cancer.

FOXM1 is a member of FOX transcription factor family 1 and is shown to be related to the process of cell viability and considered as one key prey in carcinogenic pathway. New studies indicated that FOXM1 participated in drug resistance, canceration and metastasis of tumors 52-54, so inhibiting FOXM1 may be a promising tactic for tumor treatment. Previously, several researches have shown that FOXM1 is overexpressed in multiple cancers, such as breast cancer, ovarian cancer, colon cancer, liver cancer, pancreatic cancer, ovarian cancer and gastric cancer 55. FOXM1 is modulated by carcinogenic signals, comprising lots of key factors, such as p53. Nevertheless, no studies towards FOXM1-induced effects and mechanism in ovarian cancer were shown. Our data revealed that FOXM1 expression level was raised in ovarian cancer and interlinked to the degree of malignancy and poor survival of patients with ovarian cancer. Further experiments verified that FOXM1 impelled cell proliferation and suppressed cell apoptosis. *In vitro* and *in vivo* experiments revealed that reduced FOXM1 level can rescue the effects induced by increased circular PVT1 on tumor viability. Our literature identified the mechanism of FOXM1 in tumor genesis 37.

Despite our study's promising findings, there were still limitations. The functions of circular PVT1 were explored based on *in vitro* assays in this study. The further confirmation *in vivo* will be performed in the future study. In addition, the bioinformatics analysis revealed circular PVT1 multiple pathways, including regulating angiogenesis, regulation of cell growth, regulation of apoptotic process, cell migration, and type 2 immune response. In this study, we focused on confirming the effects of Circular PVT1 on cell proliferation and migration. In the future study, we will pay attention on angiogenesis and type 2 immune response. Finally, we will collect more clinical samples and detected the expression levels of circular PVT1, miR-149-5p and FOXM1 in OV using RT-PCR assay to further confirm the clinical significance of these genes.

In conclusion, circular PVT1 increased FOXM1 level via binding to miR-149-5p and thus affected ovarian cancer cell viability, apoptosis and drug resistance.

## Abbreviations

PVT1: plasmacytoma variant translocation 1
OV: ovarian cancer
EOC: epithelial ovarian cancer
miRNAs: microRNAs
WT: wild-type
Mut: mutated
SD: standard deviation
ATCC: American Type Culture Collection
TCGA: The Cancer Genome Atlas
FOXM1: Forkhead Box Protein M1
RFS: relapse-free survival
OS: overall survival

## References

[1] Di Lorenzo GG, Ricci GM (2018). Severini, et al. Imaging and therapy of ovarian cancer: clinical application of nanoparticles and future perspectives. Theranostics.

[2] Reid BM, Permuth JB, Sellers TA (2017). Epidemiology of ovarian cancer: a review. Cancer Biol Med.

[3] Gong YB, Zou YF (2019). Clinical significance of lncRNA FAM83H-AS1 in ovarian cancer. Eur Rev Med Pharmacol Sci.

[4] Taki MK, Abiko T (2018). et al. Snail promotes ovarian cancer progression by recruiting myeloid-derived suppressor cells via CXCR2 ligand upregulation. Nat Commun.

[5] Nuti SV, Mor G, Li P (2014). TWIST and ovarian cancer stem cells: implications for chemoresistance and metastasis. Oncotarget.

[6] Moga MA, Balan A, Dimienescu OG (2019). Circulating miRNAs as Biomarkers for Endometriosis and Endometriosis-Related Ovarian Cancer-An Overview. J Clin Med.

[7] Deb B, Uddin A, Chakraborty S (2018). miRNAs and ovarian cancer: An overview. J Cell Physiol.

[8] Boyerinas B, Park SM, Murmann AE (2012). Let-7 modulates acquired resistance of ovarian cancer to Taxanes via IMP-1-mediated stabilization of multidrug resistance 1. Int J Cancer.

[9] Wendle A, Keller D, Albrecht C (2011). Involvement of let-7/miR-98 microRNAs in the regulation of progesterone receptor membrane component 1 expression in ovarian cancer cells. Oncol Rep.

[10] Liu J, Wu X, Liu H (2016). Expression of microRNA-30a-5p in drug-resistant and drug-sensitive ovarian cancer cell lines. Oncol Lett.

[11] Yu H, Y (2014). Lu, Z. Li, et al. microRNA-133: expression, function and therapeutic potential in muscle diseases and cancer. Curr Drug Targets.

[12] Qu C, Dai C, Guo Y (2019). Long noncoding RNA SNHG15 serves as an oncogene and predicts poor prognosis in epithelial ovarian cancer. Onco Targets Ther.

[13] Chen Y, Du H, Bao L (2018). LncRNA PVT1 promotes ovarian cancer progression by silencing miR-214. Cancer Biol Med.

[14] Yang Q, Yu Y, Sun Z (2018). Long non-coding RNA PVT1 promotes cell proliferation and invasion through regulating miR-133a in ovarian cancer. Biomed Pharmacother.

[15] Holdt LM, Kohlmaier A, Teupser D (2018). Molecular roles and function of circular RNAs in eukaryotic cells. Cell Mol Life Sci.

[16] Li Y (2019). Circular RNA ITCH: A novel tumor suppressor in multiple cancers. Life Sci.

[17] Song T, Xu A, Zhang Z (2019). CircRNA hsa_circRNA_101996 increases cervical cancer proliferation and invasion through activating TPX2 expression by restraining miR-8075. J Cell Physiol.

[18] Sheng R, Li X, Wang Z (2020). Circular RNAs and their emerging roles as diagnostic and prognostic biomarkers in ovarian cancer. Cancer Lett.

[19] Shabaninejad Z, Vafadar A, Movahedpour A (2019). Circular RNAs in cancer: new insights into functions and implications in ovarian cancer. J Ovarian Res.

[20] Teng F, Xu J, Zhang M (2019). Comprehensive circular RNA expression profiles and the tumor-suppressive function of circHIPK3 in ovarian cancer. Int J Biochem Cell Biol.

[21] Gu C, Huang Z, Chen X (2020). TEAD4 promotes tumor development in patients with lung adenocarcinoma via ERK signaling pathway. Biochim Biophys Acta Mol Basis Dis.

[22] Gu C, Shi X, Huang Z (2020). A comprehensive study of construction and analysis of competitive endogenous RNA networks in lung adenocarcinoma. Biochim Biophys Acta Proteins Proteom.

[23] Carithers LJ, Moore HM (2015). The Genotype-Tissue Expression (GTEx) Project. Biopreserv Biobank.

[24] Consortium GT (2013). The Genotype-Tissue Expression (GTEx) project. Nat Genet.

[25] Wang W, Zhou R, Wu Y (2019). PVT1 Promotes Cancer Progression via MicroRNAs. Front Oncol.

[26] Tang J, Li Y, Sang Y (2018). LncRNA PVT1 regulates triple-negative breast cancer through KLF5/beta-catenin signaling. Oncogene.

[27] Sarver AL, Murray CD, Temiz NA (2016). MYC and PVT1 synergize to regulate RSPO1 levels in breast cancer. Cell Cycle.

[28] Wan L, Sun M, Liu GJ (2016). Long Noncoding RNA PVT1 Promotes Non-Small Cell Lung Cancer Cell Proliferation through Epigenetically Regulating LATS2 Expression. Mol Cancer Ther.

[29] Cui D, Yu CH, Liu M (2016). Long non-coding RNA PVT1 as a novel biomarker for diagnosis and prognosis of non-small cell lung cancer. Tumour Biol.

[30] Lu D, Luo P, Wang Q (2017). lncRNA PVT1 in cancer: A review and meta-analysis. Clin Chim Acta.

[31] Wang C (2020). C-Myc-activated long non-coding RNA PVT1 enhances the proliferation of cervical cancer cells by sponging miR-486-3p. J Biochem.

[32] Chang QQ, Chen CY, Chen Z (2019). LncRNA PVT1 promotes proliferation and invasion through enhancing Smad3 expression by sponging miR-140-5p in cervical cancer. Radiol Oncol.

[33] Yan M, Gao H, Lv Z (2020). Circular RNA PVT1 promotes metastasis via regulating of miR-526b/FOXC2 signals in OS cells. J Cell Mol Med.

[34] Ghetti M, Vannini I, Storlazzi CT (2020). Linear and circular PVT1 in hematological malignancies and immune response: two faces of the same coin. Mol Cancer.

[35] Sun X, Luo L, Gao Y, Circular RNA PVT1 enhances cell proliferation but inhibits apoptosis through sponging microRNA-149 in epithelial ovarian cancer J Obstet Gynaecol Res. 2020; 46(4): 625-635.

[36] Umemori M, Kurata M, Yamamoto A (2020). The expression of MYC is strongly dependent on the circular PVT1 expression in pure Gleason pattern 4 of prostatic cancer. Med Mol Morphol.

[37] Liu YY, Zhang LY, Du WZ (2019). Circular RNA circ-PVT1 contributes to paclitaxel resistance of gastric cancer cells through the regulation of ZEB1 expression by sponging miR-124-3p. Biosci Rep.

[38] Zhu Y, Liu Y, Xiao B (2019). The circular RNA PVT1/miR-203/HOXD3 pathway promotes the progression of human hepatocellular carcinoma. Biol Open.

[39] Adhikary J, Chakraborty S, Dalal S (2019). Circular PVT1: an oncogenic non-coding RNA with emerging clinical importance. J Clin Pathol.

[40] Wang Z, Su M, Xiang B (2019). Circular RNA PVT1 promotes metastasis via miR-145 sponging in CRC. Biochem Biophys Res Commun.

[41] Qin S, Zhao Y, Lim G (2019). Circular RNA PVT1 acts as a competing endogenous RNA for miR-497 in promoting non-small cell lung cancer progression. Biomed Pharmacother.

[42] Hu J, Han Q, Gu Y (2018). Circular RNA PVT1 expression and its roles in acute lymphoblastic leukemia. Epigenomics.

[43] Chen J, Li Y, Zheng Q (2017). Circular RNA profile identifies circPVT1 as a proliferative factor and prognostic marker in gastric cancer. Cancer Lett.

[44] Zhong R, Chen Z, Mo T (2019). Potential Role of circPVT1 as a proliferative factor and treatment target in esophageal carcinoma. Cancer Cell Int.

[45] He T, Li X, Xie D (2019). Overexpressed circPVT1 in oral squamous cell carcinoma promotes proliferation by serving as a miRNA sponge. Mol Med Rep.

[46] Verduci L, Ferraiuolo M, Sacconi A (2017). The oncogenic role of circPVT1 in head and neck squamous cell carcinoma is mediated through the mutant p53/YAP/TEAD transcription-competent complex. Genome Biol.

[47] Jian Z, Ma Y Tian T (2020). Maimendong and Qianjinweijing Tang (Jin formula) suppresses lung cancer by regulation of miR-149-3p. J Ethnopharmacol.

[48] Sun Y, Liu T, Xian L (2020). B3GNT3, a Direct Target of miR-149-5p, Promotes Lung Cancer Development and Indicates Poor Prognosis of Lung Cancer. Cancer Manag Res.

[49] Sanchez-Gonzalez I, Bobien A, Molnar C (2020). miR-149 Suppresses Breast Cancer Metastasis by Blocking Paracrine Interactions with Macrophages. Cancer Res.

[50] Zhang M, Gao D, Shi Y (2019). miR-149-3p reverses CD8(+) T-cell exhaustion by reducing inhibitory receptors and promoting cytokine secretion in breast cancer cells. Open Biol.

[51] Lin RJ, Lin YC, Yu AL (2010). miR-149* induces apoptosis by inhibiting Akt1 and E2F1 in human cancer cells. Mol Carcinog.

[52] Fei BY, He X, Ma J (2017). FoxM1 is associated with metastasis in colorectal cancer through induction of the epithelial-mesenchymal transition. Oncol Lett.

[53] Shi C, Zhang Z (2017). MicroRNA-320 suppresses cervical cancer cell viability, migration and invasion via directly targeting FOXM1. Oncol Lett.

[54] Zhang J, Chen XY, Huang KJ (2016). Expression of FoxM1 and the EMT-associated protein E-cadherin in gastric cancer and its clinical significance. Oncol Lett.

[55] Liao GB, Li XZ, Zeng S (2018). Regulation of the master regulator FOXM1 in cancer. Cell Commun Signal.

